# Effects of temperature and browning on the functional response of a freshwater top predator

**DOI:** 10.1111/1365-2656.70233

**Published:** 2026-03-16

**Authors:** Viktor Nilsson‐Örtman, Erik Nilsson, Christer Brönmark

**Affiliations:** ^1^ Department of Environmental and Life Sciences Karlstad University Karlstad Sweden; ^2^ Department of Biology Lund University Lund Sweden

**Keywords:** browning, climate change, functional responses, predation, temperature, trophic cascades, visual conditions

## Abstract

1. Freshwater lakes are becoming warmer and browner, with poorly known ecosystem consequences. A major unresolved issue is how these changes will affect the feeding rates of predators that regulate top‐down trophic cascades.
2. We explored the effects of temperature and browning on the functional response and feeding rates of a keystone predator, the Northern pike *Esox lucius*.
3. We first derived a simple mechanistic model on the effect of temperature and browning on predator feeding rates. To test predictions from the model, we performed two laboratory experiments where we estimated pike functional responses in brown and clear water at three temperatures and quantified feeding rates along a gradient from completely clear to extremely brown.
4. We find strikingly weak effects of temperature and browning on pike feeding rates, even under extreme levels of browning. Pike showed an asymptotic Type II functional response under most conditions but switched to a dome‐shaped Type IV functional response in cold clear water, possibly due to seasonal changes in the schooling behaviour of prey.
5. Our results suggest that temperature and browning may have interactive effects on predator functional responses mediated via changes in prey behaviour and support the view that browning affects piscivorous fish mainly through bottom‐up effects rather than changes in foraging efficiency.

## Graphical abstract

This study explores for the first time how temperature and browning affect the functional response of a freshwater piscivore, the northern pike. The authors find surprisingly weak effects of browning, challenging visual foraging theory. Pike displayed a rare dome‐shaped functional response in cold clear water, potentially driven by seasonal changes in the behaviour of prey.

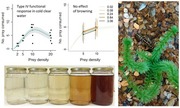

## 1 INTRODUCTION

Freshwater organisms are currently exposed to multiple stressors driven by rapid environmental change. Climate change results in rising temperatures that directly affect the vital rates of freshwater organisms (Gilbert et al., 2014). At the same time, many lakes are becoming browner due to increased concentrations of dissolved organic carbon and iron due to climate change effects (precipitation and temperature), acidification and land‐use changes (Kritzberg et al., 2020; Škerlep et al., 2020). The combination of these stressors will have important and complex effects on freshwater organisms. Increasing temperatures tend to alter metabolic rates and feeding differently, leading to changes in growth, survival and body size of individual organisms and, further, to changes in demographic rates and trophic interaction strengths (Amarasekare, 2015; Uszko et al., 2017; Vasseur & McCann, 2005). Browning, on the other hand, reduces light availability with negative effects on primary producers resulting in negative bottom‐up effects on consumer growth and biomass (Karlsson et al., 2009; Van Dorst et al., 2020). Browning also deteriorates visual conditions, which may alter feeding rates of visually oriented predators (Jönsson et al., 2011) with ecological consequences for the structure and stability of freshwater food webs. Comparable reductions in foraging performance have been reported under increased turbidity (Ortega et al., 2020), consistent with the idea that visually oriented predators are sensitive to deteriorated visual conditions. Predatory fish regulate top‐down trophic cascades in freshwater lakes by feeding on zooplanktivorous fish (Carpenter & Kitchell, 1996; Schmitz et al., 1997; Terborgh & Estes, 2013). Functional losses of top predators in shallow, clear water lakes have been associated with dramatic shifts to alternative turbid states dominated by zooplanktivorous fish and phytoplankton (Brönmark & Weisner, 1992; Scheffer & Carpenter, 2003). Our ability to predict such consequences thus critically relies on understanding how temperature and browning affect the feeding rates of freshwater piscivores.

Visual foraging theory has been developed to model the effects of deteriorated visual conditions on ecological dynamics in freshwater lakes (Clark & Levy, 1988; Hansen & Beauchamp, 2015; Rohan et al., 2021). A central assumption of this framework is that feeding rates of fish are constrained by their reaction distance: the maximum distance at which they can visually detect and react to prey. Most freshwater piscivores detect their prey using a contrast‐based visual system (De Robertis et al., 2003), and browning strongly reduces the distance at which predatory fish can detect prey (Jönsson et al., 2013; Ranåker et al., 2012). Visual foraging models thus predict dramatic declines in predator feeding rates with browning. This, however, may not always be the case, as predator feeding rates not only depend on the absolute performance of the predator but also on the relative performance of predator and prey. One of the best examples of this comes from studies of temperature‐dependent foraging in pike, *Esox lucius*. When pike forage on brown trout, *Salmo trutta*, a cold water specialist, predation rates decline precipitously below 11°C due to a shift in relative swimming performance of pike and trout at lower temperatures (Öhlund et al., 2015). A similarly abrupt change in the efficacy of predators might be associated with browning if there is a shift in the relative visual performance of predator and prey below a certain visibility threshold. Hence, understanding effects of climate change on freshwater food webs requires quantifying predator feeding rates under realistic temperature and browning scenarios.

Functional responses represent our most reliable tool for incorporating consumer feeding rates into population and community level models. These are empirically estimated functions that describe how per capita feeding rates vary with prey density. The most commonly used model is Holling's type II functional response. For predators, it describes how predator feeding rates (*F*) vary with prey density (*N*) as $F=\frac{\text{aN}}{\left(1+\mathrm{ahN}\right)},$ where *a* is attack rate, a measure of a predator's hunting efficiency, *h* is handling time (time/prey), while the inverse of handling time, 1/h, represents the predator's maximal intake rate at satiating prey densities. Other forms of functional responses are possible but rarer, including linear Type I, sigmoidal Type III or dome‐shaped Type IV responses (Holling, 1961; Jeschke et al., 2004). Theory suggests that even relatively small changes in functional response parameters can have dramatic consequences for food web stability and persistence (Gilbert et al., 2014; Vasseur & McCann, 2005). The parameters of the functional response have mechanistic interpretations that can be directly transferred to visual foraging models. Attack rate *a* can thus be expressed as the product of prey encounter rate and capture success, while encounter rate is the product of predator reaction distance squared and the relative body velocity of predator and prey, overlaid on the density and distribution of prey, all of which can be affected by visual conditions and temperature (DeLong, 2021; Pawar et al., 2012).

Functional response estimates are extremely rare for freshwater piscivores. Out of 510 experimental studies of predator functional responses reviewed by Uiterwaal et al. (2022), only 10 involved piscivorous fish, including five from freshwater lakes (Alexander et al., 2015; Cuthbert et al., 2020; Czesny et al., 2001; Galarowicz & Wahl, 2005; Wasserman et al., 2016) and five from marine habitats (Anderson, 2001; Buckel & Stoner, 2000; Harborne, 2012; Johnson, 2006; Wright et al., 1993). However, it is clear that piscivore functional responses vary in both parameters and shape depending on factors such as habitat complexity, conspecific density and body size ratios (Alexander et al., 2015; Anderson, 2001; Buckel & Stoner, 2000). To our knowledge, no studies have estimated the effect of temperature or visual conditions on the functional response of any freshwater piscivore.

Here, we explore the effect of temperature and browning on the functional response and feeding rates of a freshwater top predator, the northern pike (*E. lucius* L.), feeding on an abundant generalist zooplanktivore, roach (*Rutilus rutilus* L.). Pike is an obligate piscivore that occurs in lakes, rivers and brackish habitats throughout the Holarctic (Bregazzi & Kennedy, 1980; Nilsson & Brönmark, 2000). It is widely regarded as a keystone species that regulates top‐down trophic cascades in European temperate lakes by feeding on soft, shallow‐bodied cyprinids such as roach and common bream (*Abramis brama* Cuvier), that can constitute more than half of the total fish biomass in European lakes (Carpenter & Kitchell, 1996; Scheffer & Carpenter, 2003). Roach form dense schools in shallow littoral areas and the upper pelagic and feed exclusively on zooplankton early in life, later switching to a more diverse diet that includes zooplankton, macroinvertebrates, algae and plants (Estlander et al., 2010).

Winters have often been regarded as a quiescent period in temperate freshwater lakes. While the majority of temperate ectotherms are most active in summer, there is increasing awareness that many temperate lakes undergo dramatic changes in the spatial and temporal distribution of organisms at all trophic levels in winter (Salonen et al., 2009). The two species studied here represent a striking example of such dynamics. In many lakes, over 80% of the roach population migrate from lakes to surrounding streams in the fall and these migrations are driven by a trade‐off between foraging opportunities and predation risk by pike (Brönmark et al., 2008). The timing of these migrations is very abrupt and tends to occur when the temperature falls below ca. 8°C (Skov et al., 2010). It has been hypothesized that these abrupt migrations are caused by a shift in relative swimming performance of pike and roach at low temperatures (Hansen et al., 2020; Temple & Johnston, 1997). That is, that pike gain a substantial performance advantage over roach at temperatures below a certain thermal threshold. Resolving the mechanistic basis for these observations requires studying predator–prey interactions within the thermal window in which they occur. Here, we first formalize this hypothesis using a simple mechanistic model, derive testable predictions for the effect of temperature and browning on pike feeding rates, and then test these predictions in foraging experiments conducted under conditions experienced by pike and roach from the onset of autumn through winter into early spring.

### 1.1 A mechanistic model on the effects of temperature and browning on pike‐roach interactions

**FIGURE 1:**
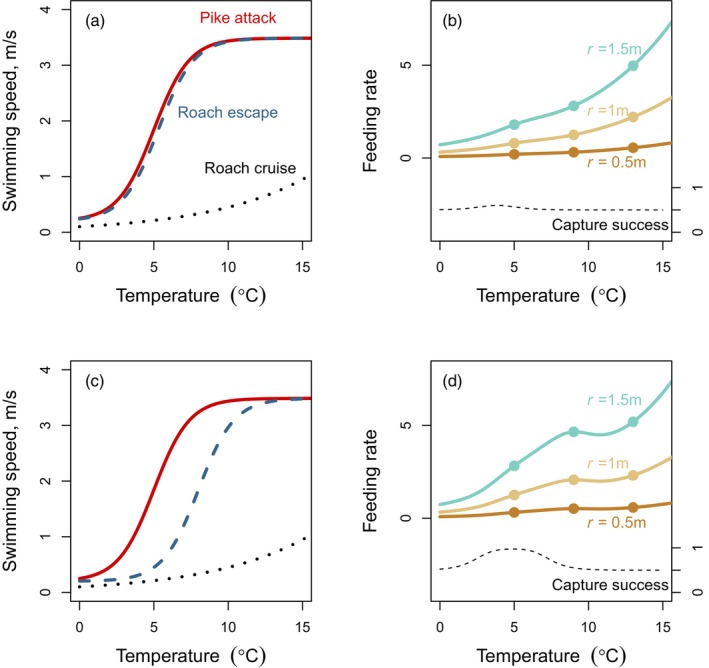
Effects of temperature and browning on predator feeding rates when predator and prey differ in relative swimming performance at low temperatures. (a, b) If temperature affects the swimming performance of predator and prey similarly, predator capture success is nearly constant across temperature (b, dashed line), and feeding rates increase monotonically with temperature. Browning (blue and brown lines in b) reduce feeding rates overall due to reductions in predator reaction distance (*r*), and lower the slope of the temperature dependence of predator feeding rates. (c, d) If the predator outperform prey at low temperatures, capture success is highest at the temperature where differences in performance is greatest (d, dashed line), and feeding rates show a local maximum at intermediate temperatures. The local maximum for feeding rate is higher than the optimum temperature for capture success, as feeding rates are the product of encounter rate (which increase with temperature) and capture success.

We here derive a simple mechanistic model describing the effects of temperature and browning on the outcome of predator–prey interactions between pike and roach. The model is based on the approach developed by Öhlund et al. (2015), but specifically adapted for pike and roach, and extended to incorporate the effects of browning. We define predator feeding rates as the product between encounter rate and capture success. Encounter rate is given by $E=A\sqrt{v_{p}^{2}+v_{r}^{2}}$, where *A* is predator search area and *v*_*p*_ and *v*_*r*_ are cruising speeds of predator and prey in m/s (Pawar et al., 2012). Search area is given by $A={\mathrm{\pi r}}^{2}$, where *r* is the reaction distance of the predator in meters (Pawar et al., 2012). As pike are predominantly sit‐and‐wait predators, we set *v*_*p*_ to 0. To account for browning, we varied *r* from 1.5 m in clear water, to 1 m in moderately brown, and 0.5 m in heavily brown water. The chosen range of reaction distances was based on empirically estimated reaction distances for 0+ pike (the age class used in our subsequent experiments) across a realistic range of browning levels (Abs_420/5_ from 0.01 to 0.40; Ranåker et al., 2012). We model the cruise speed of prey as a shallow exponential function of temperature *T*, *v*_*r*_ = 0.1*e* ^0.15*T*^, as roach cruise speed are only weakly temperature‐dependent below 15°C (Linløkken et al., 2010; Persson, 1986). We assume that predator capture success (*S*) depend on the difference in between predator attack speed (*v*_pa_) and prey escape speed (*v*_re_). Specifically, we model capture success as $S=\frac{1}{1+e^{-2\;v_{\text{diff}}}}$, where *v*_diff_ = *v*_pa_ *− v*_re_. This describes a sigmoid function where success rate is 0.5 when performance is equal, and approach 1 when the predator outperform prey. This function is consistent with data on pike capture success when foraging on cyprinids (Jolles et al., 2022). Based on data from Öhlund et al. (2015), we model the temperature dependence of pike attack speed as $v_{\mathrm{pa}}=0.2+\frac{3.2}{1+e^{\left(b-T\right)/1.2}}$, where *b* represents a temperature threshold where 50% performance is reached. For pike, we set *b* = 5 (Figure 1a). The temperature dependence of roach escape speed *v*_re_ is not known. Therefore, we assumed that its overall shape was similar to that of pike, and that differences arise from changes in the parameter *b*, which shifts the prey performance curve horizontally relative to that of the predator (as illustrated in Figure 1c).

We use this model to explore two scenarios with respect to the relative swimming performance of predator and prey. First, when the performance of predator and prey is closely matched (Figure 1a; *b*_pike_ = 5; *b*_roach_ = 5.3), predator feeding rates increase with temperature due to increases in encounter rate with increasing roach cruise speed (Figure 1b). Second, when the predator outperforms prey at lower temperatures (Figure 1c; *b*_pike_ = 5; *b*_roach_ = 8), capture success is highest at 5°C, where differences in swimming performance (*v*_diff_) are greatest (Figure 1d, dashed line). However, feeding rates show a local maximum at 9°C (Figure 1d, solid lines) as feeding rates are the product of encounter rate (that increases with temperature) and capture success. In both scenarios, browning causes marked decreases in feeding rates (Figure 1b,d) and a decrease in the slope of the temperature dependence.

Based on our mechanistic model, we make the following predictions: (1) browning will have a strong negative effect on pike attack rates and feeding rates due to reductions in reaction distance; (2) attack rates will increase with temperature in both brown and clear water; (3) browning will reduce the slope of the temperature dependence of attack rates; and (4) the temperature dependence of attack rates will be convex‐up in clear water, and near‐linear in brown water, reflecting a pike‐roach performance mismatch at low temperatures (Figure 1d). To test these predictions, we performed two laboratory experiments with young‐of‐the‐year (0+) pike and roach. We first estimated pike functional responses at three typical winter temperatures in clear and moderately brown water. Second, we estimated pike feeding rates at two prey densities along a browning gradient from completely clear to extremely brown water. Because the ecological relevance of these experimental temperatures depends on how frequently such conditions are encountered in nature, we additionally quantified the seasonal thermal regime of pike and roach across their latitudinal range using climatic simulations and used these simulations to interpret our findings in the context of present and future climate conditions.

## 2 MATERIALS AND METHODS

### 2.1 Experiment 1

We quantified functional responses of pike feeding on roach in a fully factorial foraging experiment with three temperatures (5°C, 9°C, 13°C), two visual conditions (clear vs. moderately brown water) and four prey densities (2, 5, 10, 20 roach) in 700 L arenas (wetted volume 121 cm length × 104 cm width × 56 cm depth). The temperature and browning levels reflect conditions typical of October–April in clear and brown water lakes in southern Sweden. All 24 treatment combinations were replicated eight times (192 trials). Trials were run continuously in eight temporal blocks, each block containing all 24 treatment combinations once; within blocks, the 24 trials were randomized and blocks were executed sequentially. Each trial lasted 23.5 h on average, preceded by a 15 min acclimation period. We used 24 pike divided into four groups of six. One group was used per day, and groups were rotated such that each individual was reused exactly every fourth day. Thus, pike experienced a standardized 4‐day starvation period at 9°C between successive trials. On each day, the six pike in the active group were randomly assigned to the six trials run that day.

Pike were caught by electrofishing in Lake Krankesjön in January 2023. After transport to Lund University, pike were anaesthetized, pit‐tagged and acclimated to laboratory conditions for 10–13 days at 9°C in two 700 L holding tanks with filtered and aerated water. Each tank was divided into two compartments using a perforated acrylic screen, with six pike on each side. Pike were fed once 8–10 days before trials started to encourage feeding. A total of 24 pike were used (mean wet weight 51.8 ± 12.8 g, mean ± 1 SD, *n* = 24, range: 34.5–79.2 g). Based on published length‐weight data for pike, this corresponds to fork lengths between 165 and 220 mm, which is typical for the 0+ age class in winter (Mann, 1976; Nilsson et al., 2023). Pike were used for up to 10 foraging trials (average = 8). One individual escaped after one foraging trial; its data were retained, and it was replaced by a pike that had been held under identical conditions prior to the experiment.

Prey fish were caught by electrofishing in Lake Krankesjön and connected streams on four occasions in January and February 2023. Only cyprinids from the smallest size fraction were used (wet weight = 0.91 ± 0.34 g, mean ± 1 SD, *n* = 47; fork length = 49.7 ± 5.86 mm, mean ± 1 SD, *n* = 52). These are typical sizes for the 0+ age class in winter (Mann, 1973, 1997). Roach represented more than 95% of prey used, but a small number of morphologically similar cyprinids, in particular rudd (*Scardinius erythrophthalmus* L.) and common bream, were also included. Prey fish were kept at 9°C in a 700 L holding tank with filtered and aerated water and fed live *Daphnia* and carp pellets. Newly caught prey fish were acclimated to laboratory conditions for at least 24 h before being used in foraging trials. To minimize the number of individuals required, prey that were alive and unharmed at the end of foraging trials were returned to the holding tank and reused in subsequent trials. Source populations had previously experienced pike predation, and water from the pike holding tank was continuously added to the prey holding tank to provide predator cues. We thus expect the effect of having previously been used in trials to be minor.

Foraging trials were performed in six fibre glass tanks with an interior dimension of 121 cm × 104 cm (length × width) filled to 56 cm with filtered and aerated water (total water volume: 704 L). The bottom of the tanks was covered with gravel. To facilitate recovery of uneaten prey, experimental tanks were fitted with custom‐made liners made from black PVC mesh (1.4 × 1.6 mm mesh size) that matched the interior dimensions of the tanks. Clamps and stones ensured that the mesh liner tightly fit to the bottom and sides of the tank. Three plastic aquarium plants (height ca. 30 cm) and ca. 1 L of gravel were randomly distributed over the bottom of the mesh liner. Tanks were situated in a greenhouse and exposed to natural daylight. The photoperiod ranged from 8.5:15.5 h light:dark at the beginning of the experiment to 11.5:12.5 h light:dark at the end. The temperature was controlled to within ±0.5°C using aquarium heat exchangers (Teco TK‐2000, Italy).

Visual conditions were manipulated by adding 3.5 g humic acid (Humintech GmbH, Germany) to three tanks. The clear water treatment had an absorbance (Abs_420/5_) of 0.020 ± 0.007 and the brown water treatment 0.097 ± 0.012 (based on six measurements per experimental tank taken weekly just before and during the experiment). The absorbance was measured weekly and adjusted if necessary. This is above the 0.06 Abs_420/5_ threshold used to define brown water lakes and within the range for natural lakes with roach and pike.

Trials were performed 7 days a week between 30 January and 10 March 2023. On each day, trials were run with pike that had fasted for exactly 4 days since their previous trial, and individual pike were randomly assigned to one of the treatments scheduled for that day. At the start of each trial, a single pike from the group scheduled for that day was transferred to the experimental tank with an aquarium net and allowed to acclimate for 15 min, after which 2, 5, 10 or 20 roach were added and the trial commenced. The near‐logarithmic series of prey densities was chosen following the recommendations of Uszko et al. (2020). After ca. 23.5 h, the predator was removed, the mesh liner raised, and remaining prey fish were recovered and counted. There was no disturbance from other activities in the room during the foraging trials. Due to the dark background conditions and dim natural light, no observations of foraging activity were possible. Trials were initiated around 2 PM each day and lasted on average 23.5 ± 0.96 h (mean ± 1 SD; range: 20.5–26.0 h). Pike are infrequent feeders and exhibit marked crepuscular feeding peaks at dusk and dawn (Baktoft et al., 2012). Trials were therefore run across a full diel cycle to include these natural feeding windows and integrate realized intake over time. Only fully consumed prey was attributed to predation. Nonpredation mortality was rare except for a single trial, where 7 out of 20 roach were found dead, while one had been consumed. We excluded this trial.

### 2.2 Experiment 2

In a second fully factorial feeding experiment, we quantified pike consumption across a gradient of browning levels. We performed foraging trials at two prey densities (5 and 10 roach) and five visual levels. Each treatment was replicated eight times (80 trials). As in Experiment 1, trials were run with pike that had fasted for exactly 4 days since their previous trial. Trials were run in eight temporal blocks, with each treatment combination represented exactly once in each temporal block, and with individual pike randomly assigned to one of the treatments scheduled for each day. Visual conditions were modified by adding humic acid. The absorbance in the browning treatments was 0.02 ± 0.01, 0.08 ± 0.02, 0.24 ± 0.04, 0.84 ± 0.10 and 3.06 ± 0.22 (mean Abs_420/5_ ± 1 SD; 7 weekly measurements per treatment). The two brownest treatments represent extreme levels of browning, likely limiting the visual range of pike to a few centimetres at most. In contrast to our first experiment, we thus expect feeding rates to be strongly affected by differences in encounter rates in our second experiment. The temperature was kept constant at 9°C. Pike were fasted for 12 days between Experiment 1 and 2. Two individuals were excluded as they were infested by fungi during the fasting period. The mean wet weight of pike used was 53.6 ± 12.48 g (mean ± 1 SD; *n* = 22, range: 36.6–79.2 g). Trials were performed 7 days a week between 22 March and 6 April 2023.

The experiments in this study were performed under permission from the Malmö/Lund authority for ethics of animal experimentation (Licence 5.8.18‐00783/2022).

### 2.3 Data analysis

Before fitting functional response models, we assessed whether predator body mass, experimental time or cumulative intake influenced prey consumption independently of the experimental treatments (Li et al., 2018; Schröder et al., 2016). Linear models showed that both mass and experimental day influenced consumption and that their effects varied across prey densities. To ensure this did not bias our results, we adjusted all observations to a common reference state (mean predator mass and midpoint of the experiment) using this confounder model. The full analysis is presented in Appendix 1 in the Supporting Information. Functional response models fitted to the raw and the adjusted data yielded equivalent qualitative results; we report results based on the adjusted data for consistency. Inspection of the data revealed three trials where the adjusted feeding rate was negative. These trials were performed at the beginning of the experiment and involved pike that consumed 0 out of 10, 0 out of 20 and 1 out of 20 prey, respectively. We interpret these as individuals that had not yet started to feed in the laboratory, and excluded these data points from further analysis. This did not qualitatively alter the outcome of subsequent analyses.

We determined the shape of functional responses using model selection. A set of five candidate models was tested for each treatment, including Holling's Type II, Holling's Type III and three versions of the Type IV functional responses, based on Hassell et al. (1977), Tostowaryk (1972) and Líznarová and Pekár (2013) (Appendix 2 in the Supporting Information). Models were fitted using *nls*, and compared based on their AIC scores. For treatments best described by a Type II functional response, we estimated functional response parameters (attack rate and maximum intake rate, i.e. 1/h) using the Random Predator model, which is equivalent to Holling's Type II functional response (Equation 1), but accounts for prey depletion:

(1) $$N_{T}=N_{o}-\frac{W\left(\mathrm{ah}N_{0}e^{\left(-a\left(\mathrm{TC}-hN_{0}\right)\right)}\right)}{\mathrm{ah}},$$

where *N*_0_ is initial prey density, *W* is the Lambert‐*W* function used to solve this equation (Bolker et al., 2013), *a* is attack rate and *h* is handling time. The random predator model was fitted with the *frair_fit* function in the R package *frair* using the *flexpnr* model (Pritchard et al., 2017). We calculated 95% confidence intervals for parameter estimates and model predictions using parametric bootstrapping with the *frair_boot* function from 1000 random samples. Model parameters with nonoverlapping 95% CIs were interpreted as statistically different. One treatment was best described by a dome‐shaped Type IV response. For this type of response, estimates of attack rate and maximum intake rate are not directly comparable to those of other forms of functional responses. To facilitate comparisons, we estimated attack rate, *a*, by fitting Equation (1) to data from the three lowest prey densities for this treatment; and estimated maximum intake rate (1/h) as the maximum predicted value ±95% prediction values from the best‐fitting Type IV model (i.e. the peak of the Type IV functional response), but note that these should not be interpreted as quantitative parameter estimates. Where a dome‐shaped functional response was observed, we used targeted post‐hoc linear models to compare feeding rates in clear and brown water at the two highest prey densities to aid interpretation of the fitted functional response.

Data from Experiment 2 were analysed using linear mixed models with water colour, prey density, experimental day and predator wet mass as fixed effects and pike identity as a random effect. Water colour and prey density were treated as categorical variables and day and mass were treated as continuous covariates. An interaction between prey density and water colour was included in the initial model but was not significant ($\chi_{4}^{2}$ = 3.55, *p* = 0.47) and removed from the final model. Significance of fixed effects was assessed using Type‐II Wald *χ* ^2^ tests with the *ANOVA* function in the *car* package (Fox et al., 2013). Full model output is presented in Appendix 3 in the Supporting Information. All analyses were performed in R 4.3.1 (R Core Team, 2023).

### 2.4 Thermal regime of roach and pike

We quantified the thermal regime experienced by pike and roach across their European range using the lake model FLake (Mironov, 2008; Nilsson‐Örtman et al., 2012). We ran the model with environmental forcing data from representative areas at 45° N, 50° N, 55° N, 60° N and 65° N. To validate the simulations, we surgically implanted biosensors in six adult pike and recorded their body temperature for 12 months in three large experimental ponds. The simulations and validation approach are described in detail in Appendix 4 in the Supporting Information.

## 3 RESULTS

**FIGURE 2:**
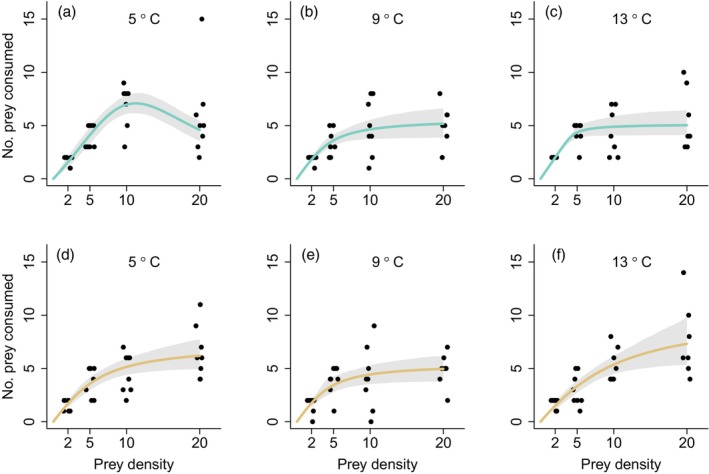
Functional responses of pike feeding on roach in clear (a–c) or moderately brown (d–f) water at three temperatures (5°C: a, d; 9°C: b, e; 13°C: c, f). Lines are predictions from best fit models for each treatment (Type IV in [a] and Type II with prey depletion in other panels). Shaded areas represent 95% confidence intervals. Data points are adjusted for body mass and time. Horizontal jitter has been added to visualize overlapping data points.

Functional responses of pike feeding on roach were best described by a Type II functional response in five out of the six treatments as judged by AIC scores (Figure 2; Appendix 5 in the Supporting Information). However, in these treatments alternative models received similar support (ΔAIC ≤2), indicating uncertainty in the precise shape of the functional response. Because prey were depleted at the lowest density in many trials, the attack‐rate estimates are associated with considerable uncertainty. We therefore report these estimates but do not interpret their absolute magnitude further. In one treatment, 5°C clear water, the functional response was best described by a dome‐shape Type IV functional response (Figure 2a). There was one apparent outlier in this treatment with strikingly high consumption at the highest prey density, but the best‐fitting Type IV model had the lowest AIC score of all models with or without this outlier (ΔAIC Type IV vs. Type II = 16.9 without outlier, ΔAIC = 2.96 with outlier). Without the outlier, the functional response in cold, clear water was best described by a Tostowaryk Type IV functional response with attack rate *a* = 0.73, handling time *h* = −0.04 and inhibition parameter *c* = 0.0005. Note that because dome‐shaped functional responses never approach a handling‐time‐limited asymptote, negative handling‐time estimates are not unexpected and should not be interpreted biologically as in Holling's classical models. The dome‐shaped response was mainly driven by increased consumption at intermediate densities, as prey consumption was higher in clear than in brown water at the second highest prey density (*F*_1,14_ = 6.09, *p* = 0.03), but did not differ between brown and clear water at the highest prey density (*F*_1,14_ = 0.14, *p* = 0.71) at 5°C.

**FIGURE 3:**
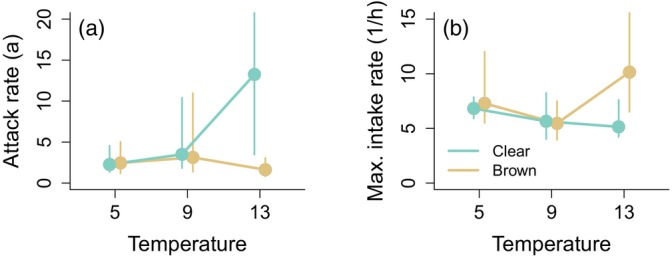
Functional response parameters attack rate (a) and maximum intake rate 1/h (b) for pike foraging on roach in clear (blue) and moderately brown (brown) water at three temperatures. Attack rates were estimated from Hollings Type II functional response with prey depletion. For the treatment with a Type IV response (5°C, Clear water), attack rate was estimated using data from the three lower prey densities, and maximum feeding rate was estimated as the maximum predicted intake rate at an intermediate prey density. Error bars show 95% CIs estimated by parametric bootstrapping for Type II functional responses and 95% prediction intervals for the Type IV maximum intake rate. Note that the upper attack rate estimate at 13°C clear water (237) falls outside the plot area.

Temperature and visual conditions had weak effects on attack rates and maximum intake rates, with some indication that the temperature response differed between water colours (Figure 3). In clear water, attack rate estimates increased with temperature in a concave‐up manner, whereas in brown water they were nearly temperature‐independent. Accordingly, confidence intervals broadly overlapped at 5°C and 9°C but were narrowly separated at 13°C (Figure 3a). Maximum intake estimates declined slightly with temperature in clear water and showed no consistent temperature trend in brown water (Figure 3b), with overlapping confidence intervals across all treatments. Note that the maximum intake rate at 5°C clear water (where a Type IV response was detected) was defined as the maximum expected number of prey consumed, corresponding to peak of the fitted curve (Figure 2a).

**FIGURE 4:**
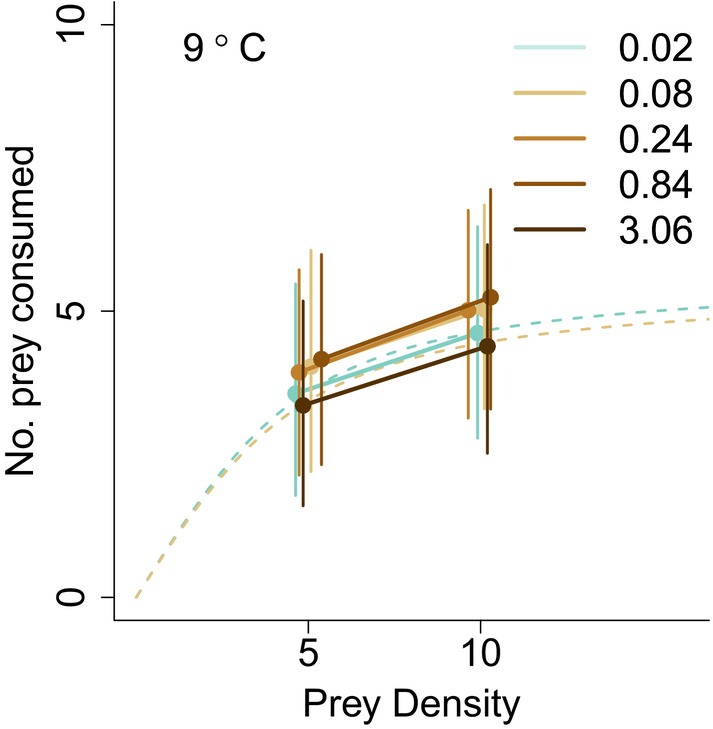
Pike prey consumption (number of prey per day) at two prey densities under five levels of visual conditions ranging from completely clear to extremely brown. Shown are predictions from a mixed effects model normalized to a pike at the midpoint of the experiment. Error bars show 95% prediction intervals. Dashed lines show functional responses estimated in 9°C clear and brown water in the functional response experiment for comparative purposes. Legend shows the water colour (Abs_420/5_) in each treatment. The brownest treatment represents extreme levels of browning, with a visual range of less than 5 cm.

In Experiment 2, water colour had no effect on pike prey consumption (Figure 4, overall effect; $\chi_{4}^{2}$ = 4.85, *p* = 0.30, water colour × prey density; $\chi_{1}^{2}$ = 3.55, *p* = 0.47). Prey consumption increased with prey density ($\chi_{1}^{2}$ = 12.04, *p* = 0.001) and pike wet weight ($\chi_{1}^{2}$ = 12.60, *p* < 0.001). A 10 g increase in pike wet weight increased the daily intake by 0.66 prey. Pike consumption showed a weak tendency to increase over time ($\chi_{1}^{2}$ = 4.06, *p* < 0.04), possibly reflecting changes in size, daylength or habituation.

**FIGURE 5:**
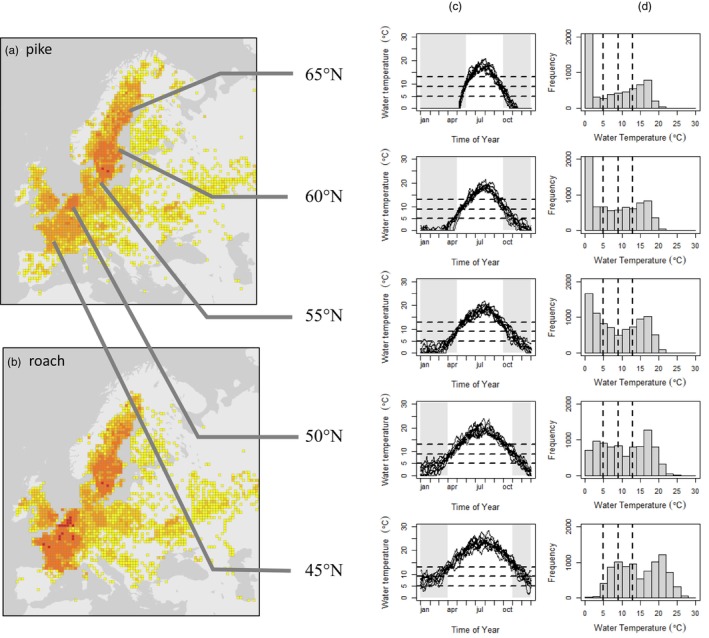
Geographic distribution and thermal regime of northern pike and roach in Europe. (a, b) Distribution of pike and roach in Europe based on records in the Global Biodiversity Information Facility GBIF. (c) Yearly temperature profiles in representative shallow lakes at different latitudes based on climatic simulations with the lake model FLake. (d) Frequency distribution of lake water temperatures at each latitude. Note that summer temperatures are relatively consistent across latitude, while the absolute amount of time spent at low and intermediate temperatures varies considerably. Dashed lines show temperatures used in the functional response experiment.

Climatic simulations showed how the thermal regime of pike and roach varies across their distribution range (Figure 5c,d). The relative duration of water temperatures was distinctly bimodal at all latitudes. The relative duration of intermediate‐to‐warm temperatures (10°C–25°C) was relatively similar across latitude, whereas the relative duration of cold (<5°C) temperatures varied strikingly. At 45° N, water temperatures below 5°C were exceedingly rare, whereas at 55° N and above, temperatures below 5°C represented the overwhelmingly most frequent conditions. In contrast to northern populations, southern populations experienced periods of temperatures above 25°C in most years, but only during brief periods of time. Data from implanted biosensors were consistent with the simulated seasonal thermal profiles (Figure S1). These simulations show that the thermal conditions under which the functional response experiment was conducted correspond to those most frequently encountered by central and northern populations during the colder half of the year.

## 4 DISCUSSION

Freshwater top predators can strongly influence lake food webs through top‐down control (Carpenter & Kitchell, 1996; Scheffer & Carpenter, 2003). Yet the functional responses of piscivores—and how they are altered by climate‐related stressors—remain largely unexplored. Here we quantified how temperature and browning affect the functional response and feeding rate of a keystone predator, the northern pike. Contrary to expectations from visual foraging theory, we found weak effects of both temperature and browning, with only a narrow set of conditions (cold, clear water) producing a qualitatively different, dome‐shaped functional response.

Our most striking result is the weak effect of browning on pike functional responses and feeding rates along a gradient from completely clear to extremely brown water. A key tenet in functional response theory and visual foraging models is that feeding rates are limited by predator reaction distance (Clark & Levy, 1988; DeLong, 2021; Hansen & Beauchamp, 2015; Pawar et al., 2012). Our mechanistic model shares this assumption and, as a result, predicts that even small reductions in reaction distance with browning should lead to severe reductions in predator feeding rates. In contrast, we find weak effects of browning on functional responses; and, most importantly, that feeding rates were completely unaffected even by the extreme levels of browning used in our second experiment.

Our first experiment included relatively modest levels of browning, slightly above the threshold for brown water lakes. Earlier experiments have shown that this level of browning significantly reduces the reaction distance of pike (Ranåker et al., 2012) and pike‐roach encounter rates and capture success (Jönsson et al., 2013). However, given the moderate level of browning and the relatively small size of the arenas, we consider it likely that encounter rates were only weakly affected by browning in the functional response experiment. Accordingly, the fitted functional response parameters from this experiment should not be interpreted as strict quantitative estimates but rather as qualitative indications of the direction and relative magnitude of effects.

In contrast, our second experiment included truly extreme levels of browning that should have severely limited the reaction distance of pike to a few centimetres, at most, in the brownest treatment, thereby strongly reducing encounter rates. It is important to notice that the two prey densities used in this experiment lie within the initial, density‐dependent, phase of the predator functional response, as determined in Experiment 1 (c.f. solid vs. dashed lines in Figure 4). If browning affects the attack rate *a* of the functional response, we would thus expect marked reductions in both the elevation and the slope of the relationship between feeding rates and prey density in this second experiment. In contrast, we find that browning had no effect on the relationship between feeding rate and prey density, despite dramatic reductions in reaction distance, emphasizing the weak effect of browning on pike foraging rates. This stands in stark contrast to meta‐analytic evidence from turbidity experiments where light attenuation consistently reduces prey capture in fish predators (Ortega et al., 2020). Even if the weak effects we observe were unexpected in the light of those results, they are consistent with field studies indicating that browning affect piscivorous fish mainly through changes in resource availability (i.e. a bottom‐up effects), rather than changes in individual foraging efficiency (Van Dorst et al., 2020). This discrepancy may reflect differences in the optical properties of brown‐coloured and turbid water. In turbid water, visual degradation mainly results from light scattering, which reduces contrast between prey and background. Browning via dissolved organic matter, on the other hand, primarily reduces light intensity, which may preserve short‐range contrast relevant for sit‐and‐wait predators such as pike (Ranåker et al., 2012). The effects of visual degradation may also depend on how evenly matched predator and prey are in their sensory and attack‐escape capabilities. Many turbidity experiments involve fish predators feeding on invertebrate prey, where feeding rates are primarily constrained by prey detection rather than capture or escape dynamics (Ortega et al., 2020). In contrast, interactions between piscivorous fish and fish prey involve predator and prey with comparable sensory modalities and effective escape behaviours. In such matched systems, visual degradation may affect predator and prey in ways that partially cancel out at the level of realized feeding rates. Together, these differences point to strong context dependence in how visual degradation influences feeding rates.

Although our attack‐rate estimates should be interpreted cautiously, the functional response experiment nevertheless suggested an interaction between browning and temperature: attack rates increased with temperature in clear water but were essentially flat in brown water. This pattern is consistent with the qualitative prediction from our mechanistic model that browning dampens the temperature dependence of attack rate. In our model, this arises because the larger search area in clear water amplifies temperature‐driven increases in relative body velocities. In our experiment, encounter rates were likely only weakly limited by reaction distance, so the observed pattern may instead reflect browning effects on activity itself. Many fish, including pike and roach, reduce movement in brown water (Jönsson et al., 2013). Attack rates may thus have increased with temperature in clear water due to higher movement of one or both species, whereas low activity at all temperatures in brown water kept attack rates unchanged.

Surprisingly, we find that pike switched from a monotonic functional response under most conditions to a dome‐shaped Type IV functional response in cold, clear water. To our knowledge, this represents the first documented case of a dome‐shaped functional response in a freshwater piscivorous fish. Outside this treatment, alternative monotonic models received similar support (ΔAIC ≤2), indicating that the exact functional response shape under most conditions cannot be identified with confidence. Although we only observed a Type IV functional response under a very specific set of environmental conditions (clear water at 5°C), our climatic simulations suggest that pike and roach currently experience these exact conditions during a large part of the year in many shallow temperate lakes, implying that the behavioural regime under which Type IV responses emerged is not rare but rather one of the most frequently experienced in nature. Even moderate levels of warming and browning could thus lead to major changes in the frequency with which local populations experience these conditions. We note that in deeper, stratified lakes, behavioural thermoregulation may decouple fish body temperature from the temperature of the open water column, so the thermal regimes derived here should be interpreted as specific to shallow lake systems. Nevertheless, biosensor records from free‐ranging pike were consistent with the simulated seasonal thermal regime for shallow lakes, supporting the relevance of these simulations for interpreting winter predator–prey interactions. Theory suggests that Type IV responses can destabilize predator–prey dynamics and drive predators to extinction (Köhnke et al., 2020). As we only observed Type IV responses under a restricted set of conditions, we consider it unlikely that negative density‐dependent predation rates would directly influence the persistence of pike and roach populations. Instead, we suggest that the existence of such effects may help understand how freshwater fish segregate in space and time over the year, described next.

Type IV responses are rare, but have often been associated with predators that capture prey that engage in social group defence, including schooling behaviour (Jeschke & Tollrian, 2005; Líznarová & Pekár, 2013). Schooling is ubiquitous in aquatic ecosystems (Maury, 2017; Shaw, 1978) and has been shown to reduce predator capture success due to the so‐called ‘swarming effect’ (Jeschke & Tollrian, 2007), although results for pike and roach have been mixed (Neill & Cullen, 1974; Turesson & Brönmark, 2007). Even when swarming effects are strong, dome‐shaped responses can be notoriously difficult to detect in experiments as these often require prey densities and arenas much larger than those typically used in functional response experiments (Jeschke & Tollrian, 2005). These challenges are further exacerbated when studying large‐bodied piscivores. In the light of this, it is notable that Type IV responses have previously been observed in at least one other piscivore, the coral reef mesopredator graysby (*Cephalopholis cruentata* Lacepède). This species shifts from a Type II functional response in summer to a Type IV functional response in winter, likely due to a stronger schooling behaviour of prey in winter (Harborne, 2012). In our study, the conditions that gave rise to a Type IV functional response coincide with the conditions experienced by pike and roach at the onset of winter, when a large proportion of the adult roach population migrate to surrounding streams, and 0+ roach gather in massive aggregations in sheltered microhabitats. Based on this, we suggest that Type IV responses may be a relatively common but underappreciated feature of piscivore functional responses, and that they may be associated with predators that feed on prey that display seasonal changes in their tendency for social aggregation. We recommend that future functional response experiments with piscivores should be designed specifically with swarming effects in mind and routinely test for dome‐shaped functional responses.

Our results provide limited support for the idea that roach experience greater predation risk at low temperatures due to a shift in the relative swimming performance of pike and roach. If this was the case, we predicted attack rates to show a convex‐up temperature dependence, especially in the clear water treatment. In contrast, attack rates showed a concave‐up temperature dependence in clear water and a flat temperature response in brown water. We note, however, that the highest predation rates overall occurred at intermediate prey densities in cold, clear water. This raises the possibility that pike did, in fact, have higher capture success during encounters with small groups of roach in cold, clear water, but that this did not affect the temperature dependence of functional response parameters. To explore this further will require quantifying the effects of temperature and browning on the components of the predator foraging cycle (body velocities, reaction distance, attack rate, capture success) as well as the functional response itself under realistic optical and thermal conditions. More definitive tests will likely require experiments in arenas large enough to keep encounter rates limiting even at moderate levels of browning.

Our results reinforce the view that winters are a dynamic time in freshwater lakes. Traditionally, it has been assumed that temperate lakes come to an ecological standstill in the winter. More recently, however, it has become clear that this is very far from the truth (Salonen et al., 2009). Recent tracking studies have revealed that piscivores such as pike remain strikingly active throughout winter (Baktoft et al., 2012) and that their prey display a remarkable diversity in their winter behavioural strategies (Jepsen & Berg, 2002). Our study contributes to our understanding of these dynamics by showing that temperature and browning can have interactive effects on the functional response of a freshwater apex predator, possibly due to seasonal changes in the behaviour of its prey, and highlights the need to understand how warmer winters and browner water will affect predator–prey interactions in temperate freshwater lakes.

## AUTHOR CONTRIBUTIONS

Viktor Nilsson‐Örtman and Christer Brönmark conceived the ideas and designed the methodology; Viktor Nilsson‐Örtman and Erik Nilsson collected the data; Viktor Nilsson‐Örtman developed the theoretical model, analysed the data and led the writing of the manuscript. All authors contributed critically to the drafts and gave final approval for publication.

## CONFLICT OF INTEREST STATEMENT

The authors declare no competing interests.

## Supporting information


**Appendix S1.** Adjustment for body size and time.
**Appendix S2.** Fitted functional response models.
**Appendix S3.** Analysis of data from Experiment 2.
**Appendix S4.** Climate simulations of the thermal regime of roach and pike.
**Appendix S5.** Functional response model comparison results.

## Data Availability

Data and code available from the Dryad Digital Repository https://doi.org/10.5061/dryad.kkwh70sjc (Nilsson‐Örtman et al., 2026).
