# Supplementary material for: Effects of temperature and browning on the functional response of a freshwater top predator
Source: J Anim Ecol. 2026 Mar 16;95(5):824–36. doi: 10.1111/1365-2656.70233 (PMC13145305; doi:10.1111/1365-2656.70233)
Supplement: Supplementary file 1 — Appendix S1. Adjustment for body size and time. Appendix S2. Fitted functional response models. Appendix S3. Analysis of data from Experiment 2. Appendix S4. Climate simulations of the thermal regime of roach and pike. Appendix S5. Functional response model comparison results. [file JANE-95-824-s001.docx]

| **Appendix 1. Adjustment for body size and time** Before fitting functional-response models, we evaluated whether predator body mass, experimental day or cumulative intake could bias consumption independently of the experimental treatments. To do this, we first fitted a linear model including all experimental factors (Temperature, Watercolour, Prey density) together with body mass, experimental day and cumulative intake (m_init). This model showed significant main effects of body mass and experimental day. Cumulative intake had no detectable effect and was removed. We then tested whether the effects of body mass and experimental day varied across prey densities, because such interactions would bias the estimation of attack rate or handling time. Both Prey density x Predator wet weight (p = 0.076) and Prey density x Day of Experiment (p = 0.036) showed density-dependent structure, and we therefore retained these terms in the final confounder model (m_adj). This model was then used to adjust all observations to a common predator mass (mean) and experimental day (midpoint), before fitting functional-response models. FR models fitted on raw and adjusted data yielded qualitatively identical inferences; we therefore report results based on the adjusted values to reduce known confounding effects.****Table S1.**** Type II ANOVA for the full model (m_init) including cumulative intake. | | | | |
| --- | --- | --- | --- | --- |
| **Type II ANOVA — m_init** | | | | |
| *Row* | *Sum.Sq* | *Df* | *F.value* | *Pr..F.* |
| Temperature | 14.756 | 2 | 1.491 | 0.228 |
| Watercolor | 0.007 | 1 | 0.001 | 0.970 |
| Prey density | 402.426 | 3 | 27.101 | <0.001 |
| Predator wet weight (g) | 20.165 | 1 | 4.074 | 0.045 |
| Day of Experiment | 75.168 | 1 | 15.186 | <0.001 |
| Cumulative intake | 4.385 | 1 | 0.886 | 0.348 |
| Residuals | 900.855 | 182 | NA | NA |

### ****Table S2.**** Type II ANOVA for the reduced adjustment model (m_conf), used to generate size- and time-adjusted consumption values.

| **Type II ANOVA — m_adj (final adjustment model)** | | | | |
| --- | --- | --- | --- | --- |
| *Row* | *Sum.Sq* | *Df* | *F.value* | *Pr..F.* |
| Temperature | 19.177 | 2 | 2.045 | 0.132 |
| Watercolor | 0.411 | 1 | 0.088 | 0.768 |
| Prey density | 398.195 | 3 | 28.310 | 0.000 |
| Predator wet weight (g) | 23.082 | 1 | 4.923 | 0.028 |
| Day of Experiment | 72.198 | 1 | 15.399 | <0.001 |
| Prey density x Predator wet weight (g) | 32.831 | 3 | 2.334 | 0.076 |
| Prey density x Day of Experiment | 40.914 | 3 | 2.909 | 0.036 |
| Residuals | 829.864 | 177 | NA | NA |

### ****Table S3.**** Summary of fixed effects for the reduced adjustment model (m2), showing that body mass and experimental time affected consumption more strongly at the higher prey densities.

|  | **Number of prey eaten** | | | |
| --- | --- | --- | --- | --- |
| *Predictors* | *Estimates* | *CI* | *Statistic* | *p* |
| (Intercept) | 2.401 | -0.499 – 5.302 | 1.634 | 0.104 |
| Temperature9 | -0.782 | -1.548 – -0.015 | -2.011 | **0.046** |
| Temperature13 | -0.468 | -1.248 – 0.311 | -1.185 | 0.238 |
| WatercolorClear | 0.095 | -0.535 – 0.724 | 0.296 | 0.768 |
| Prey density 5 | 1.483 | -2.492 – 5.458 | 0.736 | 0.463 |
| Prey density 10 | 2.581 | -1.595 – 6.756 | 1.220 | 0.224 |
| Prey density 20 | 1.275 | -3.018 – 5.568 | 0.586 | 0.559 |
| Predator wet weight (g) | -0.010 | -0.061 – 0.041 | -0.372 | 0.711 |
| Day of Experiment | 0.016 | -0.065 – 0.096 | 0.391 | 0.696 |
| Prey density 5 x Predator wet weight (g) | 0.020 | -0.050 – 0.089 | 0.557 | 0.578 |
| Prey density 10 x Predator wet weight (g) | 0.045 | -0.026 – 0.117 | 1.252 | 0.212 |
| Prey density 20 x Predator wet weight (g) | 0.093 | 0.020 – 0.167 | 2.495 | **0.014** |
| Prey density 5 x Day of Experiment | -0.034 | -0.141 – 0.073 | -0.629 | 0.530 |
| Prey density 10 x Day of Experiment | -0.093 | -0.189 – 0.004 | -1.892 | 0.060 |
| Prey density 20 x Day of Experiment | -0.127 | -0.224 – -0.029 | -2.562 | **0.011** |
| Observations | 192 | | | |
| R^2^ / R^2^ adjusted | 0.390 / 0.342 | | | |
| AIC | 857.916 | | | |

**Appendix 2. Fitted functional response models.**

Holling’s Type II: $F=\frac{aN}{(1+ahN)}$

Holling’s type III: $F=\frac{aN^{k}}{\left( 1+ahN^{k} \right)}$

Hassell’s Type IV: $F=\frac{aN^{2}}{(1+ahN^{2}+cN)}$

Tostowaryk’s Type IV: $F=\frac{aN}{(1+ahN+acN^{3})}$

Líznarová & Pekár’s Type IV: $F=\frac{aN}{(1+ahN+acN^{2})}$

In the Type III model, *k* describes the slow initial rise of the sigmoidal response. In Type IV models, c is an ‘inhibition factor’ describing the effect of prey defense at high densities.

**Appendix 3. Analysis of data from Experiment 2.**

Prey consumption in Experiment 2 was analyzed using linear mixed-effects models. Fixed effects included water color, prey density, experimental day, and predator wet mass; individual pike (PIT tag) was included as a random intercept to account for repeated measures. Water color and prey density were modelled as categorical factors, whereas day and mass were included as continuous covariates. An initial model including the interaction between water color and prey density showed no significant interaction (Table S4), and this term was removed from the final model. In the final model, water color had no detectable effect on consumption, whereas prey density, experimental day, and predator mass were significant predictors (Table S5). Model estimates (±SE), test statistics, AIC, variance components, and marginal/conditional R² are reported in Table S6.

| **Table S4. Type II ANOVA — Full model (with interaction)** | | | |
| --- | --- | --- | --- |
| *Term* | *Chisq* | *Df* | *Pr..Chisq.* |
| Watercolor | 4.833 | 4 | 0.305 |
| Prey density | 11.863 | 1 | 0.001 |
| Day of experiment | 3.681 | 1 | 0.055 |
| Predator wet mass (g) | 15.607 | 1 | <0.001 |
| Watercolor x Prey density | 3.550 | 4 | 0.470 |

| **Table S5. Type II ANOVA — Final model (interaction removed)** | | | |
| --- | --- | --- | --- |
| *Term* | *Chisq* | *Df* | *Pr..Chisq.* |
| Watercolor | 4.854 | 4 | 0.303 |
| Prey density | 12.042 | 1 | 0.001 |
| Day of experiment | 4.056 | 1 | 0.044 |
| Predator wet mass (g) | 12.603 | 1 | <0.001 |

**Table S6. Model estimates test statistics, AIC, variance components, and marginal/conditional R² for the final model from Experiment 2.**

|  | **Number of prey eaten** | | | |
| --- | --- | --- | --- | --- |
| *Predictors* | *Estimates* | *CI* | *Statistic* | *p* |
| (Intercept) | -0.737 | -3.062 – 1.588 | -0.632 | 0.529 |
| Watercolor: 0.1 | 0.557 | -0.402 – 1.517 | 1.159 | 0.250 |
| Watercolor: 0.3 | 0.440 | -0.537 – 1.417 | 0.899 | 0.372 |
| Watercolor: 1.0 | 0.612 | -0.425 – 1.649 | 1.178 | 0.243 |
| Watercolor: 3.5 | -0.223 | -1.211 – 0.765 | -0.450 | 0.654 |
| Prey density: 10 | 1.065 | 0.453 – 1.677 | 3.470 | **0.001** |
| Day of experiment | 0.063 | 0.001 – 0.125 | 2.014 | **0.048** |
| Predator wet mass (g) | 0.066 | 0.029 – 0.103 | 3.550 | **0.001** |
| **Random Effects** | | | | |
| σ^2^ | 1.57 | | | |
| τ_00_ _PIT_ | 0.55 | | | |
| ICC | 0.26 | | | |
| N _PIT_ | 20 | | | |
| Observations | 79 | | | |
| Marginal R^2^ / Conditional R^2^ | 0.316 / 0.495 | | | |
| AIC | 301.760 | | | |

**Appendix 4. Climate simulations of the thermal regime of roach and pike**

**4.1 FLake simulations**

We quantified the thermal environment experienced by pike and roach in shallow lakes across Europe, from Southern France (45°N) to Northern Sweden (65°N; Figure 1a). On average, the mean annual air temperature differed by 2.7°C between sites separated by 5° of latitude. This is within the range of warming predicted at each latitude by 2099 under IPCC scenario AFA1. These simulations thus both provide data on the historical thermal regime at each latitude, as well as the expected thermal regime by 2099. Lake water simulations were performed using the meteorological lake model FLake (Mironov 2008). FLake works by creating a one-dimensional (vertical) representation of a lake, divided into regions representing the mixed water layer, thermocline, bottom layer and bottom sediment layer. This model has proven highly accurate when applied to real lakes and has been used operationally for weather forecasting. We ran the model using atmospheric forcing data from the ECMWF Era-Interim dataset at 12h intervals for the period 2001–2010 (Simmons *et al.* 2007). We set the water depth to 4m, the secchi depth to 3m, and a wind fetch of 200m. This corresponds to relatively clear, small and shallow lakes. We ran the model with environmental forcing data from grids located at 45°N, 50°N, 55°N and 60°N (Figure 1a). All grids were situated near the coast to minimize impact of continental climates. Additionally, we ran simulations with reduced visual conditions (secchi depth 0.3m); this had minor effects on the thermal regime (not shown). From each simulation, we saved the estimated, twice daily, surface water temperatures and used these to characterize the historical and future thermal regime across latitude.

**References**

Mironov, D.V. (2008). Parameterization of lakes in numerical weather prediction. Description of a lake model. COSMO Technical Report.

Simmons, A., Uppala, S., Dee, D. & Kobayashi, S. (2007). ERA-Interim: New ECMWF reanalysis products from 1989 onwards. ECMWF Newsletter, 110, 25–35.

**4.2 Field body temperature of pike**

To validate the simulations, we equipped six adult pike with biosensors and recorded their body temperature for 12 months in experimental ponds. Pike were captured by electrofishing in Lake Krankesjön, Sweden, in November 2021 and transported to three experimental ponds in Vomb, Sweden (70m x 30m, depth 1.5m) where they were placed in keep nets (3 x 3 x 1.5 m, 1.5 cm mesh size; Pokorný Sítě, Brloh, Czech republic) for acclimation. Pike were surgically implanted with Centi-HRT biosensors (Star-Oddi, Garðabær, Iceland) set to record body temperatures at 30 minute intervals and released into the ponds on November 23, 2021. In early October 2022, all pike were recaptured by electrofishing, biosensors retrieved, and body temperature data downloaded. Results show a similar range in temperatures, and a distinct bimodal distribution, matching the results from the climatic simulations (Figure S1).


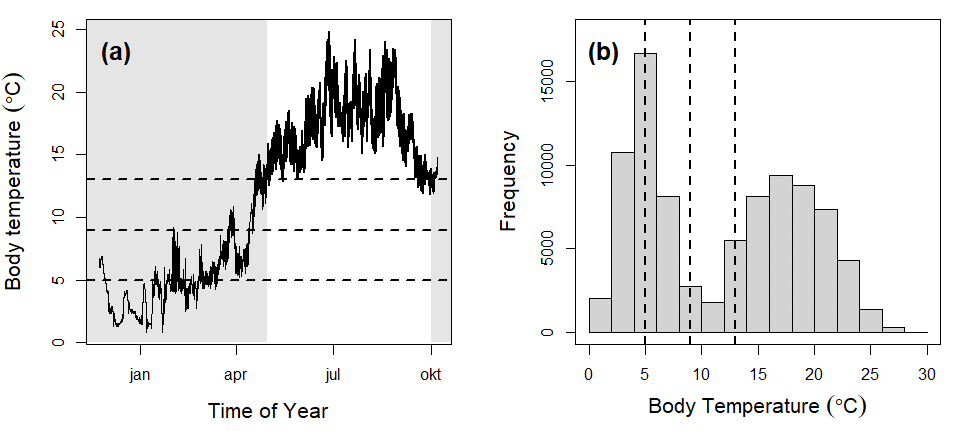


**Figure S1.** Body temperature of free-swimming pike, based on data from biosensors implanted in the body cavity of 6 adult pike in two experimental ponds in Vomb, Sweden. (A) Body temperature recordings from a representative pike individual from November 2021 to October 2022. Shaded areas represent the ‘cold season’ from October 1^st^ to April 30^th^. (B) Frequency distribution of body temperatures in 8 wild pike individuals between November 2021 and October 2022. Dashed lines show temperatures used in the functional response experiment.

**Appendix 5. Functional response model comparison results**

| **Table S7. Model comparison using AIC and Akaike weights for the 5°C clear water treatment (all observations)** | | | |
| --- | --- | --- | --- |
| *Model* | *AIC* | *ΔAIC* | *Akaike.weight* |
| Tostowaryk | 148.433 | 0.000 | 0.375 |
| Liznarova | 148.579 | 0.146 | 0.349 |
| Hassell | 150.901 | 2.468 | 0.109 |
| HollingII | 151.388 | 2.955 | 0.086 |
| HollingIII | 151.481 | 3.049 | 0.082 |

| **Table S8. Model comparison using AIC and Akaike weights for the 5°C clear water treatment (outlier removed)** | | | |
| --- | --- | --- | --- |
| *Model* | *AIC* | *ΔAIC* | *Akaike.weight* |
| Tostowaryk | 113.095 | 0.000 | 0.587 |
| Liznarova | 113.851 | 0.756 | 0.402 |
| Hassell | 121.142 | 8.047 | 0.010 |
| HollingIII | 127.440 | 14.345 | 0.000 |
| HollingII | 129.992 | 16.897 | 0.000 |

| **Table S9. Model comparison using AIC and Akaike weights for the 9°C clear water treatment** | | | |
| --- | --- | --- | --- |
| *Model* | *AIC* | *ΔAIC* | *Akaike.weight* |
| HollingII | 125.145 | 0.000 | 0.363 |
| Tostowaryk | 126.578 | 1.433 | 0.177 |
| Liznarova | 126.646 | 1.501 | 0.171 |
| HollingIII | 126.956 | 1.811 | 0.147 |
| Hassell | 127.020 | 1.875 | 0.142 |
| **Table S10. Model comparison using AIC and Akaike weights for the 13°C clear water treatment** | | | |
| *Model* | *AIC* | *ΔAIC* | *Akaike.weight* |
| HollingII | 123.988 | 0.000 | 0.392 |
| Hassell | 125.756 | 1.768 | 0.162 |
| HollingIII | 125.825 | 1.837 | 0.156 |
| Tostowaryk | 125.976 | 1.988 | 0.145 |
| Liznarova | 125.988 | 2.000 | 0.144 |

| **Table S11. Model comparison using AIC and Akaike weights for the 5°C brown water treatment** | | | |
| --- | --- | --- | --- |
| *Model* | *AIC* | *ΔAIC* | *Akaike.weight* |
| HollingII | 125.019 | 0 | 0.433 |
| Tostowaryk | 126.504 | 1.484 | 0.206 |
| Liznarova | 126.624 | 1.605 | 0.194 |
| HollingIII | 126.918 | 1.899 | 0.167 |
| Hassell | – | – | – |

| **Table S12. Model comparison using AIC and Akaike weights for the 9°C brown water treatment** | | | |
| --- | --- | --- | --- |
| *Model* | *AIC* | *ΔAIC* | *Akaike.weight* |
| HollingII | 131.708 | 0.000 | 0.341 |
| HollingIII | 132.880 | 1.172 | 0.190 |
| Hassell | 132.887 | 1.179 | 0.189 |
| Liznarova | 133.446 | 1.738 | 0.143 |
| Tostowaryk | 133.534 | 1.827 | 0.137 |

| **Table S13. Model comparison using AIC and Akaike weights for the 13°C brown water treatment** | | | |
| --- | --- | --- | --- |
| *Model* | *AIC* | *ΔAIC* | *Akaike.weight* |
| HollingII | 131.766 | 0 | 0.469 |
| HollingIII | 133.644 | 1.878 | 0.183 |
| Liznarova | 133.747 | 1.981 | 0.174 |
| Tostowaryk | 133.76 | 1.994 | 0.173 |
| Hassell | – | – | – |
